# Control of *Hes7* Expression by Tbx6, the Wnt Pathway and the Chemical Gsk3 Inhibitor LiCl in the Mouse Segmentation Clock

**DOI:** 10.1371/journal.pone.0053323

**Published:** 2013-01-09

**Authors:** Aitor González, Iris Manosalva, Tianxiao Liu, Ryoichiro Kageyama

**Affiliations:** 1 Institute for Virus Research, Kyoto University, Kyoto, Japan; 2 Japan Science and Technology Agency, CREST, Kyoto, Japan; National Cancer Center, Japan

## Abstract

The mouse segmentation is established from somites, which are iteratively induced every two hours from the presomitic mesoderm (PSM) by a system known as the segmentation clock. A crucial component of the segmentation clock is the gene *Hes7*, which is regulated by the Notch and Fgf/Mapk pathways, but its relation to other pathways is unknown. In addition, chemical alteration of the Wnt pathway changes the segmentation clock period but the mechanism is unclear.

To clarify these questions, we have carried out *Hes7* promoter analysis in transgenic mouse embryos and have identified an essential 400 bp region, which contains binding sites of Tbx6 and the Wnt signaling effector Lef1. We have found that the *Hes7* promoter is activated by Tbx6, and normal activity of the *Hes7* promoter in the mouse PSM requires Tbx6 binding sites. Our results demonstrate that Wnt pathway molecules activate the *Hes7* promoter cooperatively with Tbx6 in cell culture and are necessary for its proper expression in the mouse PSM. Furthermore, it is shown that the chemical Gsk3 inhibitor LiCl lengthens the oscillatory period of *Hes7* promoter activity.

Our data suggest that Tbx6 and the Wnt pathway cooperatively regulate proper *Hes7* expression. Furthermore, proper *Hes7* promoter activity and expression is important for the normal pace of oscillation.

## Introduction

Vertebrate embryos are divided along the longitudinal axis into somites in a process called segmentation. During the vertebrate segmentation, a new pair of bilateral somites arises every two hours from the presomitic mesoderm (PSM). The pace of somite formation correlates with the periodic expression of genes of the Notch, Fgf and Wnt pathways [1].

The *Hes7* gene, a crucial component of the segmentation clock, is downstream of the Notch and Fgf pathways and drives the oscillation of several cyclic genes of these pathways [2]–[4]. The Fgf pathway is mainly active in the posterior PSM, whereas Notch pathway activity is found in the PSM and budding somites [5], [6]. This raises the question of how the domain of *Hes7* expression is specified.

The oscillation period of the segmentation clock in vertebrates is changed after perturbation of the Notch and Wnt pathways [7]–[10]. The effect of Notch pathway perturbations on the segmentation clock period is understandable, because Notch target genes are critical components of the segmentation clock. By contrast, the mechanism of the Wnt pathway contribution to the segmentation clock period is unclear.

To investigate these questions, we have analyzed the *Hes7* promoter and have found evidence that Tbx6 and the Wnt pathway regulate *Hes7* expression in the PSM. Our results suggest that Tbx6 and the Wnt pathway are necessary for proper *Hes7* expression. We have also found that treatment with the chemical Gsk3 inhibitor LiCl activates the Wnt pathway and lengthens the oscillatory period of *Hes7* expression.

## Materials and Methods

### Plasmids

The luciferase reporters were created by inserting the 2.6 kb *Hes7* promoter (−2573, cctgcttgg… to +76, …ggaggagc) [11] into the pGL3 plasmid (Promega). Transcription factor binding sites were found using the Genomatix Suite (www.Genomatix.de). For the H7p2.6dR reporter, we introduced point mutations in the Rbpj binding sites at −124 bp (CTTaCaACAT), −425 bp (TTTaCaACAG), −467 bp (TTGTtGtAAT), and −1463 bp (GTGTtGtAAA) to generate the H7p2.6dR construct. Then different promoter sizes (H7p1.9dR: −1939 bp (tggaagac…) to +76; H7p1.4dR: −1410 (ttctctcc…) to +76; H7p1.0dR: −998 (cccaggcc…) to +76) were amplified by PCR, sequenced and subcloned into the pGL3-basic (Promega) vector to obtain the luciferase reporter. Two point mutations were introduced in the T-box binding sites at −1306 (TGCcGGTtAG) and −1350 (CCCAaAaCCG) by PCR for H7p1.4dRdT. For the lacZ reporters, the *luciferase* gene was replaced with the *lacZ* gene. The expression plasmids for NICD [11], Tbx6, T [12], human LEF1 [13] and constitutively active Ctnnb1 (S37A mutation) [14] were kind gifts of the authors.

### Transgenic embryos

Transgenic embryos were generated as previously described [15]. Embryos were genotyped and stained with X-gal using a standard protocol.

### Embryo culture, inhibitor treatments and bioluminescence imaging

Embryos were taken out and freed from extraembryonic structures in prewarmed PBS. For *in situ* hybridization assays, wild-type embryos were transferred to culture medium (DMEM, 10% FBS, 1% P/S) in the presence of solvent or inhibitor and cultured for the indicated times at 37°C with 5% CO_2_. For timelapse imaging experiments, tails of embryos were cut up to the second somite, transferred to 1 µM luciferin-containing culture medium and genotyped by observation of luminescence with a CCD camera. One positive tail was selected, transferred to inhibitor containing medium and immediately imaged (Conditions: 5% CO_2_, 85% O_2_ and 37°C). The oscillation period was measured by two different methods by analyzing movies that spanned at least two oscillation cycles. In the first method, we counted the number of oscillation cycles and divided it by their duration. In the second method, we created a spatiotemporal plot of the oscillations with time in the x-axis and measured the distance between peaks (Figure S6). Inhibitors were: 20, 40 and 100 mM LiCl (Nacarai Tesque), 10 µM Gsk3 Inhibitor IX (BIO) (EMD), 1 and 5 µM XAV939 (Sigma) and 100 µM CKI-7 (Sigma).

### Luciferase assay

The luciferase assay was carried out as previously described using C3H10T1/2 cells in 10% FBS and 1% P/S in DMEM and Lipofectamin LTX/Plus (Invitrogen) transfection reagent [11].

### Electrophoresis mobility shift assay (EMSA)

We subcloned the Tbx6 cDNA from pCS2-3xFlag-Tbx6 [12] into the T7 control plasmid of the TNT *in vitro* translation kit (Promega). The *in vitro* translation was carried out following the manufacturer's protocol. Oligonucleotides (15 bp) containing the wild-type and mutant T-box binding sites were annealed and labeled with gamma-^32^P-ATP (GE Heathcare) using T4 polynucleotide kinase. The sense-strand sequences of the wild-type and mutant probes for T-box −1306 are: agc ctc acg tgc agg tga gaa aaa ctc aac and agc ctc acg tgc Cgg tTa gaa aaa ctc aac, respectively and for T-box −1350: cag ggg cgg ccc cac acc cgg gtg caa act g and cag ggg cgg ccc caA aAc cgg gtg caa act g, respectively. Purified proteins and labeled probes were incubated for 30 min at room temperature in 5× binding buffer (100 mM Tris-HCl, pH 8.0, 300 mM KCl, 5 mM EDTA, 60% Glycerol, 3.3 mg/ml BSA, 8.35 mM DTT, 12.5 mM MgCl_2_). Samples were then electrophoresed on a 5% non-denaturing polyacrylamide gel (Acryl/bis-acryl 19∶1 ratio, 0.5× Tris/Borate/EDTA buffer, 2.5% Glycerol).

### *In situ* hybridization and real-time PCR

*In situ* hybridization was performed as previously described [2]. To quantify the *in situ* hybridizations after inhibitor culture, we classified control and treated embryos into categories of high and low staining and plotted the percentage of control and treated embryos with high staining. For real-time PCR, the PSM was cut until the first visible somite border, RNA was isolated using the NucleoSpin RNA II kit (Macherey-Nagel) and reverse transcribed with SuperScript II Reverse Transcriptase (Invitrogen). Two biological replicates were carried out. Real-time PCR was performed with the LightCycler 480 SYBR Green I Master mix and the Real-time PCR system (Roche). Primer sequences (forward and reverse) are Gapdh: atc ttc ttg tgc agt gcc agc ctc gtc ccg and agt tga ggt caa tga agg ggt cgt tga tgg; Axin2 (QT00126539, Quiagen) and Msgn1: cgg ctt agt cga gct gga tta and ctc cgc tgg aca gac atc ttg.

### Immunofluorescence detection

For immunofluorescence detection, embryos were fixed in PFA incubated overnight at 4°C, paraffin embedded and sectioned (10 µm). Antigen retrieval was carried out in citrate buffer (pH 6) placed in an autoclave at 105°C for 15 min. After 1 h blocking at room temperature, primary antibodies were diluted in Can Get Signal immunostain Solution B (1∶1000, anti-Hes7, [2]; 1∶1000, anti-Tbx6 [16]; 1∶200, anti-beta Catenin (610153, BD Biosciences) and incubated overnight. For detection, slides were incubated with Alexa antibodies (Invitrogen) and counterstained with DAPI. Images were acquired on a Zeiss LSM510 laser scanning microscope (Carl Zeiss MicroImaging).

### Statistical analysis

To calculate statistical significance, differences between control and treated populations were evaluated with the Student's t test. Error bars indicate standard error of the mean.

## Results and Discussion

### A crucial 400 bp sequence in the *Hes7* promoter

**Figure 1:**
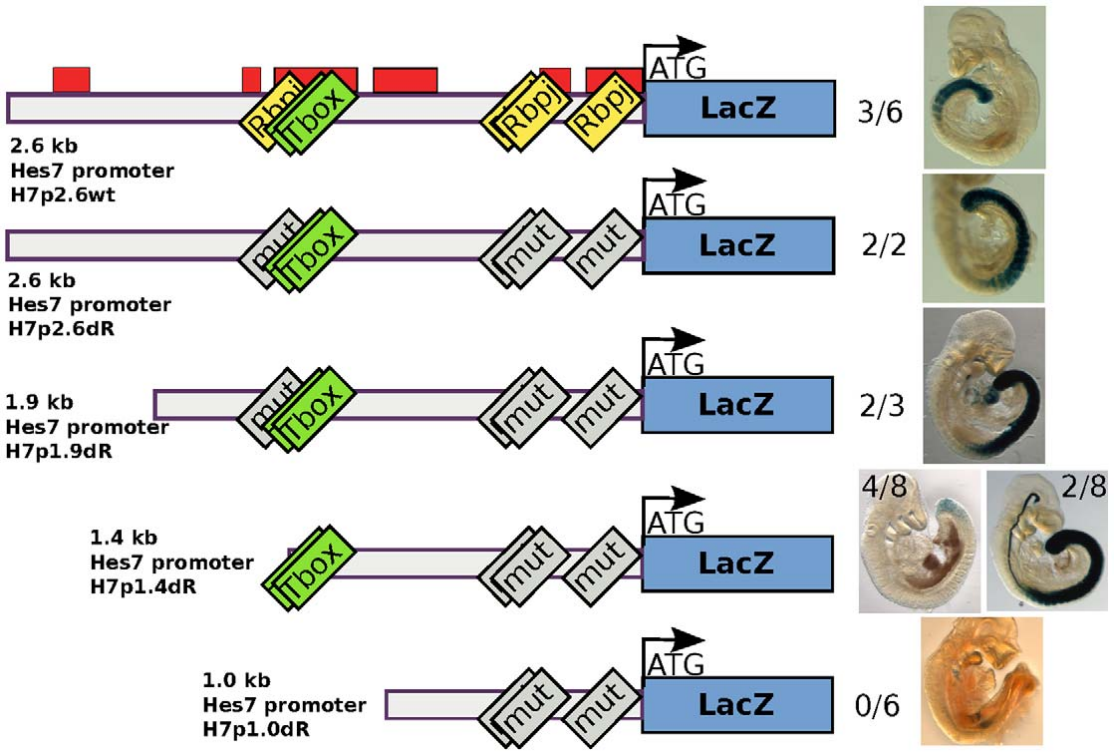
*Hes7* promoter analysis with lacZ reporter transgenic embryos. The left side is a scheme of the *Hes7* promoter constructs. From top to bottom: H7p2.6wt, H7p2.6dR, H7p1.9dR, H7p1.4dR and H7p1.0dR. The promoter size relative to the transcription start is shown. The red boxes show conserved sequences according to the Vista browser [33]. The yellow and green boxes show Rbpj and T-box binding sites, respectively, while the gray boxes stand for mutated sites. The two numbers show the number of X-gal positive embryos and transgene positive embryos, respectively.

To construct the *Hes7* promoter reporter, we linked the 2.6 kb upstream promoter region of *Hes7* (H7p2.6wt) to the *lacZ* gene. Transgenic embryos showed X-gal staining in the endogenous *Hes7* expression region and somites (Figure 1). The somitic staining was likely due to the stability of the *lacZ* mRNA and protein. We did not use destabilizing sequences, because we were mainly interested in finding promoter sequences required for the posterior onset of *Hes7* promoter activity. Previous data showed posterior *Hes7* expression in the absence of Notch signaling [4]. Therefore, we hypothesized that Rbpj binding sites are not required for the posterior onset of *Hes7* expression. To test this hypothesis, we introduced point mutations into four Rbpj binding sites (H7p2.6dR). This construct also showed X-gal staining in the posterior PSM (Figure 1). We created smaller 1.9 kb, (H7p1.9dR), 1.4 kb (H7p1.4dR) and 1.0 kb (H7p1.0dR) promoter reporters. H7p1.9dR and H7p1.4dR reporters showed X-gal staining, although H7p1.4dR showed either weak activity (4/8 embryos) or ectopic notochord X-gal staining (2/8 embryos) (Figure 1). We speculate that T (Brachyury) exerts a repressive effect on a T-box binding site in the 1.9-1.4 kb region to prevent this ectopic notochord activity. Finally, the X-gal staining of the PSM was lost in the H7p1.0dR reporter, demonstrating that this 400 bp Hes7 promoter region is important for Hes7 promoter activity (Figure 1). This 400 bp region is strongly conserved in humans and contains binding sites for interesting transcription factors such as Lef1 (Wnt pathway effector) [17], Ets (Fgf pathway effector) [18], the mesodermal factor Tbx6 [19], and the axial elongation factor Cdx2 [20] (Figure S1).

### *Hes7* promoter activity requires Tbx6 binding sites

**Figure 2:**
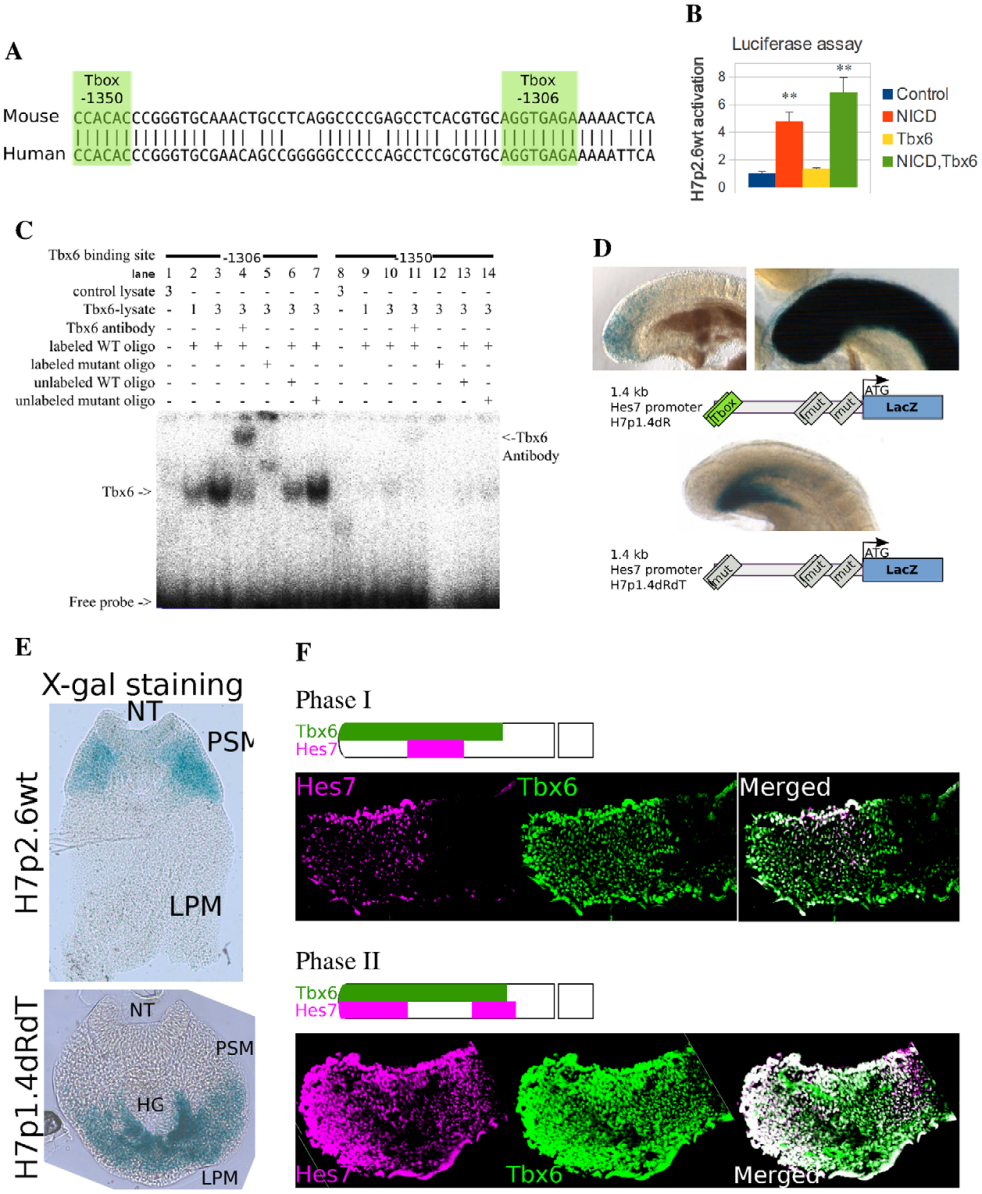
Requirement of Tbx6 for *Hes7* expression. (A) Alignment of mouse (−1350 to −1291, Top) and human (Bottom) *Hes7* promoter sequences with two T-box binding sites (T-1306 and T-1350). (B) Luciferase assay of the H7p2.6wt promoter after transfection of NICD and Tbx6 expression plasmids. The *Hes7* promoter is activated by NICD and further enhanced by Tbx6. (C) Electromobility gel shift assay (EMSA) of the T-box containing oligos and Tbx6. Tbx6 binds to T-box containing oligos. Conditions: control lysate (Lanes 1,8), low (Lanes 2,9) and high Tbx6 lysate concentration (Lanes 3–7,10–14), supershift for Tbx6 antibody (Lanes 4,11), wild-type (WT) (Lanes 2–4,6,7,9–11,13,14) and mutant labeled oligonucleotides (Lanes 5,12) and competition with unlabeled wild-type (Lanes 6,13) and mutant (Lanes 7,14) oligonucleotides. (D,E) X-gal staining of transgenic embryos with the H7p1.4dR (WT T-box sites; n = 8) and H7p1.4dRdT (mutated T-box sites; n = 2) reporter constructs and vibratome sections of control and H7p1.4dRdT reporters. Mutated T-box binding sites of the *Hes7* promoter reporter prevent the WT X-gal staining (D). Vibratome sections of control reporter constructs with WT T-box binding sites show that all positive X-gal staining embryos also present staining in the paraxial mesoderm (E top, n = 13). By contrast, reporter constructs with mutated T-box binding sites prevent paraxial mesoderm staining and induce lateral plate and ventral mesoderm staining (E bottom, n = 2). NT, neural tube; PSM, presomitic mesoderm; HG, hindgut; LPM, lateral plate mesoderm. (F) Double immunofluorescence detection of Hes7 (magenta) and Tbx6 (green) (Phase I, n = 3; Phase II, n = 2). The anterior limit of Hes7 protein coincides or slightly exceeds the Tbx6 protein domain suggesting that the *Hes7* mRNA domain is always included in the Tbx6 protein domain.

Two T-box binding sites caught our attention (T-1306 and T-1350), because such binding sites are important in other PSM gene promoters, namely *Dll1, Mesp2* and *Msgn1* (Figures 1 and 2A) [12], [21], [22]. First, we co-transfected a luciferase reporter of the wild-type (WT) 2.6 kb *Hes7* promoter (H7p2.6wt) with Notch intracellular domain (NICD) and Tbx6 expression plasmids. NICD upregulated the H7p2.6wt promoter reporter (Figure 2B). Tbx6 induced a non-significant increase of the H7p2.6wt reporter activity alone and enhanced the positive effect of NICD (Figure 2B). These data suggest that Tbx6 enhances NICD mediated activation of the *Hes7* promoter in cultured cells. This is similar to the reported enhancement of *Mesp2* reporter activity by NICD and Tbx6 [12].

Next, we checked whether *in vitro* synthesized Tbx6 can bind to oligos containing the two T-box binding sites by electrophoresis mobility shift assay (EMSA). The EMSA showed that *in vitro* synthesized Tbx6 protein can bind to WT (Figure 2C, lanes 2, 3, 9, 10), but not mutant (Figure 2C, lanes 5, 12), oligos containing these binding sites. The significance of the larger band in the presence of the mutant oligo is unclear (Figure 2C, lane 5). The WT bands were supershifted by anti-Tbx6 antibody (Figure 2C, lanes 4, 11), while they were antagonized by WT but not mutant unlabeled oligos (Figure 2C, lanes 6, 7, 13, 14). The bands were very strong for the T-1306 binding site but weak for T-1350, suggesting that Tbx6 mainly binds to the T-1306 binding site.

**Figure 3:**
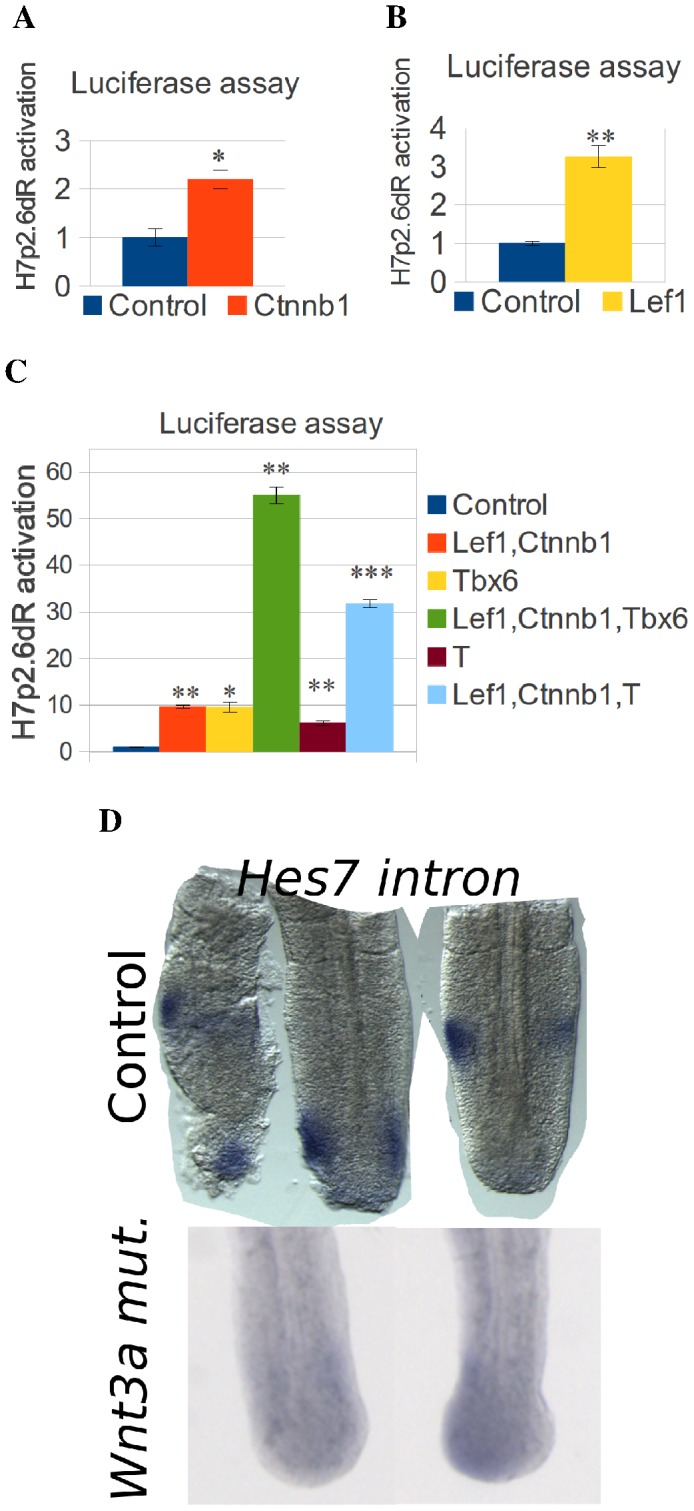
Activation of *Hes7* expression by the Wnt pathway. (A,B,C) *Hes7* promoter luciferase reporter assay of the H7p2.6dR promoter reporter after co-transfection of constitutively active *Ctnnb1*, *Lef1*, *T* and *Tbx6* expression plasmids. (A,B) The H7p2.6dR promoter reporter is activated by constitutively active *Ctnnb1* and *Lef1* expression plasmids. (C) The Ctnnb1 and Lef1 mediated activation of the H7p2.6dR promoter reporter is synergistically enhanced by co-transfection of both T and Tbx6 expression plasmids. (D) *Hes7* intronic expression in *Wnt3a* hypomorph E10.5 mutant embryos. In the mutant, *Hes7* intronic expression (n = 7) is downregulated compared to the control (n = 9).

To verify whether these T-box binding sites are also important in the PSM during embryonic development, we constructed a lacZ reporter with H7p1.4dRdT with point mutations in the two T-box binding sites of H7p1.4dR, and subsequently generated transgenic embryos. The H7p1.4dRdT lacZ reporter did not show X-gal staining in the tail bud and PSM in contrast to the control, suggesting that these T-boxes are important for WT *Hes7* expression (Figure 2D). In control embryos, the *Hes7* promoter reporter activity is found in the paraxial mesoderm; however in embryos expressing the H7p1.4dRdT lacZ contruct, X-gal staining is mainly in the lateral plate mesoderm (Figure 2E). A similar phenotype was reported for the *Msgn1* promoter in the absence of T-box binding sites [22]. These data suggest that Tbx6 is very important for the initiation of *Hes7* expression. The observation that Tbx6 alone did not significantly upregulate Hes7 in cultured cells may be due to the lack of factors that potentiate Tbx6 activity in the PSM such as the Wnt pathway (Figure 3C) [22]. To investigate whether Tbx6 also defines the anterior limit of *Hes7* expression, we examined the protein expression localization of Hes7 and Tbx6 proteins in the PSM by immunofluorescence. We found variable patterns of Hes7 protein that were mostly included within the Tbx6 protein domain (Figure 2F). Specifically, in phases II/III of Hes7 protein expression, the anterior limit of Hes7 protein coincided or slightly exceeded that of Tbx6 (Phase II in Figure 2F). *Hes7* gene is only transcribed in the Hes7 protein negative region [2], suggesting that *Hes7* transcription occurs only in the Tbx6 protein domain. These results suggest that Tbx6 is important for controlling the proper expression of *Hes7* in the PSM.

### The Wnt pathway activates *Hes7* expression

Tbx6 regulates target genes synergistically with the Wnt pathway [21], [22]. Therefore, we also investigated, whether the Wnt pathway activates the *Hes7* promoter. To this end, we co-transfected the Rbpj mutated 2.6 kb *Hes7* promoter reporter with expression plasmids of a constitutively active form of beta-Catenin (Ctnnb1) and Lef1 in C3H10T1/2 cells and analyzed the luciferase luminescence. Our results show that the *Hes7* promoter is activated by Ctnnb1 and Lef1(Figure 3A,B). Then, we co-transfected *Tbx6* and *T* expression plasmids and found that these plasmids further upregulated the activation of the H7p2.6dR construct by Ctnnb1 and Lef1 (Figure 3C). Previously, it was found that inactivation of Ctnnb1 in the PSM downregulates *Hes7* expression [23]; therefore, we analyzed the hypomorph *Wnt3a* mutant mouse *Vestigial* (*Vt*) [24]. In *Wnt3a* mutant embryos at embryonic stage E10.5, *Hes7* intronic expression was downregulated (Figure 3D). *Tbx6* mRNA expression was also downregulated in these embryos in agreement with results in PSM specific *Ctnnb1* conditional KO mice (Figure S2) [23]. These results suggest that *Hes7* expression is positively regulated by the Wnt pathway.

### LiCl lengthens the oscillation period

LiCl has been reported to activate the Wnt pathway targets by inhibiting Gsk3b, a kinase that targets Ctnnb1 for degradation [25], [26]. First we tested whether LiCl can activate the Wnt pathway in the PSM. We cultured E9.5 embryos for 6 h in the presence of 0, 20 and 40 mM LiCl, cut the PSM until the first somite and synthesized cDNA. Real-time PCR for the Wnt target genes *Axin2* and *Msgn1* showed an increased expression of these genes in the presence of LiCl (Figure S3A,B). Second, we also determined whether the Ctnnb1 protein is stabilized by LiCl in the PSM. Our results show higher levels of Ctnnb1 in the PSM of E10.5 embryos cultured with 40 mM LiCl for 6 h (Figure S3C), which led us hypothesized that LiCl may activate Hes7 expression. To test it, we cultured E10.5 WT mouse embryos with 20 mM LiCl for 6 h. Our results show a non-significant tendency to higher Hes7 expression in the presence of LiCl (Figure S4A). Culture of E9.5 embryos with a higher LiCl concentration (100 mM) also showed a non-significant tendency to higher Hes7 mRNA expression after 2 h culture (Figure S4B). Treatment of E9.5 embryos with Gsk3 Inhibitor IX for 2 h also showed a non-significant increase of Hes7 mRNA expression (Figure S4C). These results suggest that LiCl activates the Wnt pathway. The low effect of LiCl on *Hes7* expression might be due to a compensatory effect of the autoinhibitory feedback of Hes7 protein on itst promoter.

**Figure 4:**
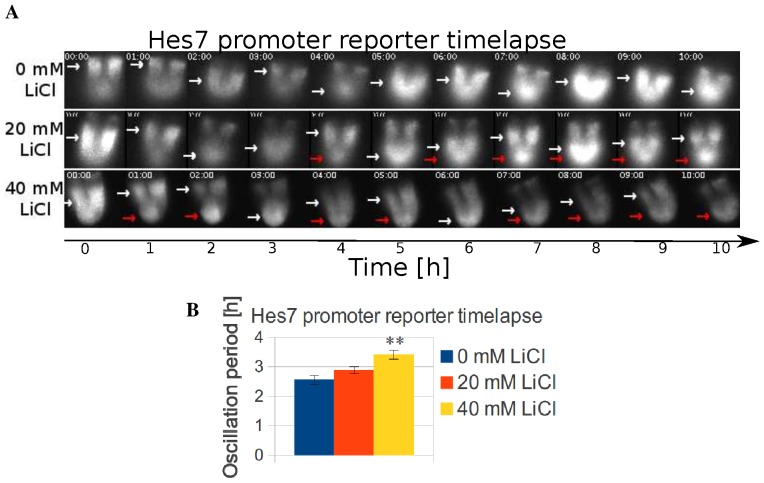
Lengthening of the oscillatory period of the *Hes7* promoter activity by LiCl. (A,B) Timelapse imaging of E10.5 *Hes7* promoter luciferase reporter embryos in the presence of 20 and 40 mM LiCl and period quantification. The control sample shows the oscillatory promoter activity bands (White arrows), while the 20 and 40 mM LiCl-treated samples show an abnormal posterior band (Red arrows) (A). Quantification shows an increase of the oscillation period in a dose-dependent manner for the control (n = 11), 20 mM LiCl (n = 12) and 40 mM LiCl (n = 4) of 2.5 h, 2.9 h and 3.6 h (B).

Therefore, we hypothesized that the effect of LiCl on *Hes7* would manifest as a change of the oscillatory period. Therefore, we monitored the oscillations of E10.5 transgenic embryos carrying a *Hes7* promoter luciferase reporter by timelapse microscopy in the presence of LiCl [15]. In the control experiment, we found luminescence bands spreading anteriorly (Figure 4A, Movie S1). In contrast, 20 and 40 mM LiCl treatments induced an abnormal band of *Hes7* promoter activity in the posterior PSM (Figure 4A, red arrows, Movies S2, S3). Furthermore, in some embryos, 40 mM LiCl treatment resulted in arrest of the oscillations of *Hes7* promoter activity by locking the reporter in an active state (Figure S5, Movie S4). Quantification of the oscillation period in the presence of LiCl demonstrated that treatment with 40 mM LiCl increased the oscillation period (Figure 4B). To exclude the possibility that the longer period was a consequence of the arrested oscillation, we determined the duration of individual cycles after addition of LiCl (Figure S6). Our results show that the largest period difference occurred during the second oscillation cycle after inhibitor addition suggesting that the period change is not a consequence of the arresting oscillation.

Our data indicate that in the PSM, as in other tissues, LiCl increases the rate of transcription of target genes through stabilization of Ctnnb1 (Figure S3). Interestingly, a higher rate of transcription might be responsible for the longer period of Hes7 promoter activity oscillations in the presence of LiCl in agreement with numerical models of Hes7 that predict a positive correlation between higher rate of transcription and longer period [27], [28].

We then hypothesized that an inhibitor of the Wnt pathway would shorten the oscillation period. The small molecule XAV939 stabilizes Axin and increases Ctnnb1 degradation [29]. Timelapse imaging of *Hes7* promoter activity with low concentration (1 µM) of XAV939 did not cause apparent effects the reporter activity, but high concentration (5 µM) downregulated promoter activity (Figure S7, Movies S5, S6). We measured the oscillation period for embryos treated with 1 µM XAV939 but did not observe any effect (Figure S7).

A previous study found a correlation between lower *Hes7* expression and longer oscillation period due to treatment with the chemical agent CKI-7, which targets the Wnt pathway [7]. That result is unclear, because to our understanding, low *Hes7* expression was observed with high concentration (200 µM) of CKI-7, whereas the longer period was found with a different lower concentration (100 µM) of CKI-7 [7]. Therefore, we also examined the oscillatory activity of the *Hes7* promoter in the presence of 100 µM CKI-7. Measurement of the oscillation period showed an increase of the period from 2.5 h to 3.3 h (Figure S8, Movie S7), which agreed with the previous data [7]. By contrast, *in situ* hybridization of embryos cultured with 100 µM CKI-7 for 3 h did not show a clear change of *Hes7* expression levels (data not shown). These results suggest that CKI-7 inhibitor lengthens the oscillatory period although the mechanism for this effect requires further investigation.

Our results suggest that the Wnt related chemicals LiCl and CKI-7 lengthen the period, whereas another Wnt related chemical, XAV939, does not. Our data that LiCl upregulates the expression of the Wnt target genes *Axin2* and *Msgn1* and the levels of Ctnnb1 suggest that the effects of LiCl on the period is mediated by the Wnt pathway. However, a mutant mouse where Ctnnb1 is stabilized does not change the oscillation period [30]. Therefore, alternative possibilities could be explored in the future. For instance LiCl could act through the Mapk pathway [31], which is also important for *Hes7* expression [32].

**Figure 5:**
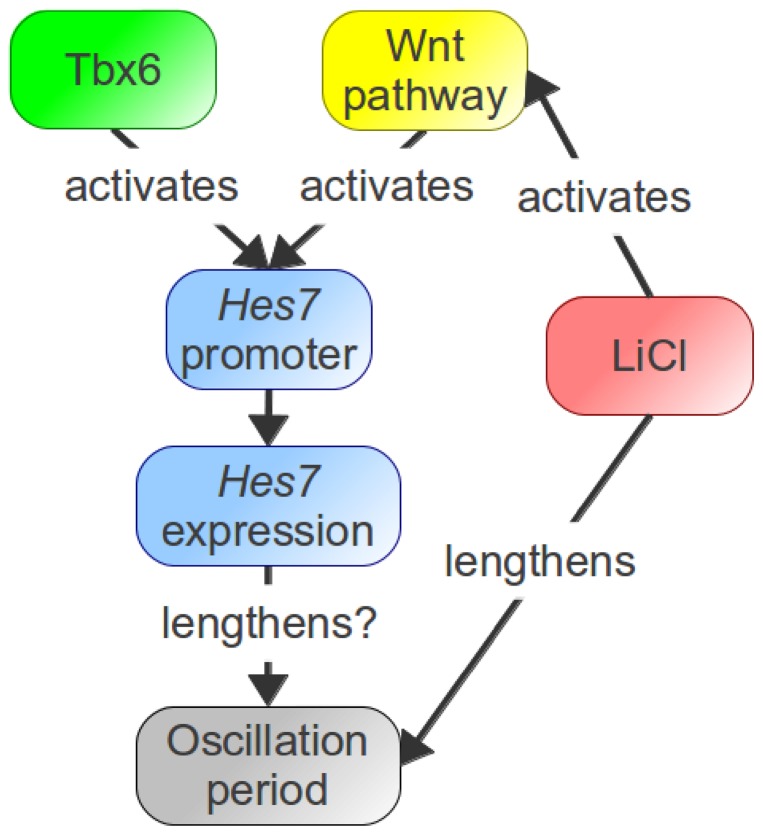
Role of Tbx6 and Wnt pathway on *Hes7* regulation. Tbx6 binding sites and the Wnt pathway are required for normal *Hes7* expression in the PSM. Furthermore, the Gsk3 inhibitor LiCl activates the Wnt pathway and lengthens the oscillatory period of *Hes7* promoter activity.

Here, we have investigated the transcriptional activation of *Hes7* expression, in an effort to better understand the mechanism of the mouse segmentation clock. We found evidence that Tbx6 binding sites are required for normal *Hes7* promoter activity (Figure 5) and also that *Hes7* expression is positively regulated by the Wnt pathway (Figure 5). Furthermore, we showed that proper Wnt activity is important for normal expression of *Hes7*, and that different Wnt-related chemicals lengthen the oscillatory period of *Hes7* promoter activity (Figure 5). Tbx6, the Wnt pathway and *Hes7* are very important molecules for the murine segmentation clock. Therefore, our analysis of their regulation provides new insights into the mechanism of the segmentation clock.

## Supporting Information

Figure S1 **Sequence alignment of the region between −1.4 and −1.0 kb of the mouse** ***Hes7*** **promoter (Top) with human (Bottom) and transcription factor binding sites (TFBS).** Several conserved binding sites of transcription factors expressed in the PSM can be found.
(TIF)

Figure S2 ***In situ*** **hybridization of** ***Tbx6*** **mRNA in E10.5** ***Wnt3a*** **mutant embryos.** Tbx6 expression is downregulated in the *Wnt3a* hypomorph embryos (Control: n = 2; Mutant: n = 2).
(TIF)

Figure S3 **LiCl activates the Wnt pathway in the PSM.** (A,B) E9.5 embryos were cultured with LiCl for 6 h and the expression of *Axin2* and *Msgn1* was measured by real-time PCR. The expression of both *Axin2* (A) and *Msgn1* (B) increases in the presence of 40 mM LiCl (0 mM: n = 10; 20 mM: n = 10; 40 mM: n = 10). (C) E10.5 embryos were cultured with 40 mM LiCl for 6 h and immunohistochemistry was carried with a Ctnnb1 antibody. Ctnnb1 protein is stabilized by 40 mM LiCl treatment (0 mM: n = 6; 40 mM: n = 8).
(TIF)

Figure S4 ***Hes7 in situ*** **hybridization of embryos cultured in the presence of Gsk3 inhibitors.** E10.5 embryos were cultured with 20 mM LiCl for 6 h (Control: n = 7; Treated: n = 10) (A), E9.5 embryos with 100 mM LiCl for 2 h (Control: n = 13; Treated: n = 14) (B) and E9.5 embryos with 10 µM Gsk3 Inhibitor IX for 6 h (Control: n = 13; Treated: n = 12) (C). Our results show a non-significant increase of the *Hes7* expression levels under these culture conditions.
(TIF)

Figure S5 **Timelapse imaging of E10.5 Hes7 promoter luciferase reporter embryos in the presence of the 40 mM LiCl.** Some embryos treated with this concentration of LiCl showed stabilization of the *Hes7* promoter reporter activity and arrest of oscillations (Red arrowheads).
(TIF)

Figure S6 **Period quantification method and duration of oscillation cycles after addition of LiCl.** To measure the oscillatory period, we created a spatiotemporal plot of the timelapse activity with time in the x-axis and measured the distance between peaks in the posterior PSM (A). This allows us to measure the duration of individual cycles after addition of the chemical LiCl. We started to observe a period difference during the second oscillation cycle (B).
(TIF)

Figure S7 **Timelapse imaging of E10.5** ***Hes7*** **promoter luciferase reporter embryos in the presence of the Wnt inhibitor XAV939 and period quantification.** (A) The control and 1 µM XAV939 samples show the normal reporter activity, while 5 µM XAV939 treatment rapidly downregulates *Hes7* promoter activity. (B) XAV939 treatment does not change the period between the control (n = 11) and the 1 µM XAV939 samples (n = 2).
(TIF)

Figure S8 **Period quantification of the oscillations of the E10.5** ***Hes7*** **promoter reporter embryos in the presence of the chemical agent CKI-7 observed by timelapse imaging.** Treatment with 100 µM CKI-7 lengthens the period from 2.5 h (n = 11) to 3.3 h (n = 3).
(TIF)

Movie S1 **Control timelapse imaging of a transgenic embryo at E10.5 carrying a** ***Hes7*** **promoter luciferase reporter.**
(AVI)

Movie S2 **Timelapse imaging of a transgenic embryo at E10.5 carrying a** ***Hes7*** **promoter luciferase reporter in the presence of 20 mM LiCl.**
(AVI)

Movie S3 **Timelapse imaging of a transgenic embryo at E10.5 carrying a** ***Hes7*** **promoter luciferase reporter in the presence of 40 mM LiCl.**
(AVI)

Movie S4 **Timelapse imaging of a transgenic embryo at E10.5 carrying a** ***Hes7*** **promoter luciferase reporter in the presence of 40 mM LiCl that shows constant promoter activity.**
(AVI)

Movie S5 **Timelapse imaging of a transgenic embryo at E10.5 carrying a** ***Hes7*** **promoter luciferase reporter in the presence of 1 µM XAV939.**
(AVI)

Movie S6 **Timelapse imaging of a transgenic embryo at E10.5 carrying a** ***Hes7*** **promoter luciferase reporter in the presence of 5 µM XAV939 that shows inactivation of the promoter activity.**
(AVI)

Movie S7 **Timelapse imaging of a transgenic embryo at E10.5 carrying a** ***Hes7*** **promoter luciferase reporter in the presence of 100 µM CKI-7 inhibitor.**
(AVI)
