# Supplementary figures and images for: Control of Hes7 Expression by Tbx6, the Wnt Pathway and the Chemical Gsk3 Inhibitor LiCl in the Mouse Segmentation Clock
Source: PLoS One. 2013 Jan 9;8(1):e53323. doi: 10.1371/journal.pone.0053323 (PMC3541138; doi:10.1371/journal.pone.0053323)

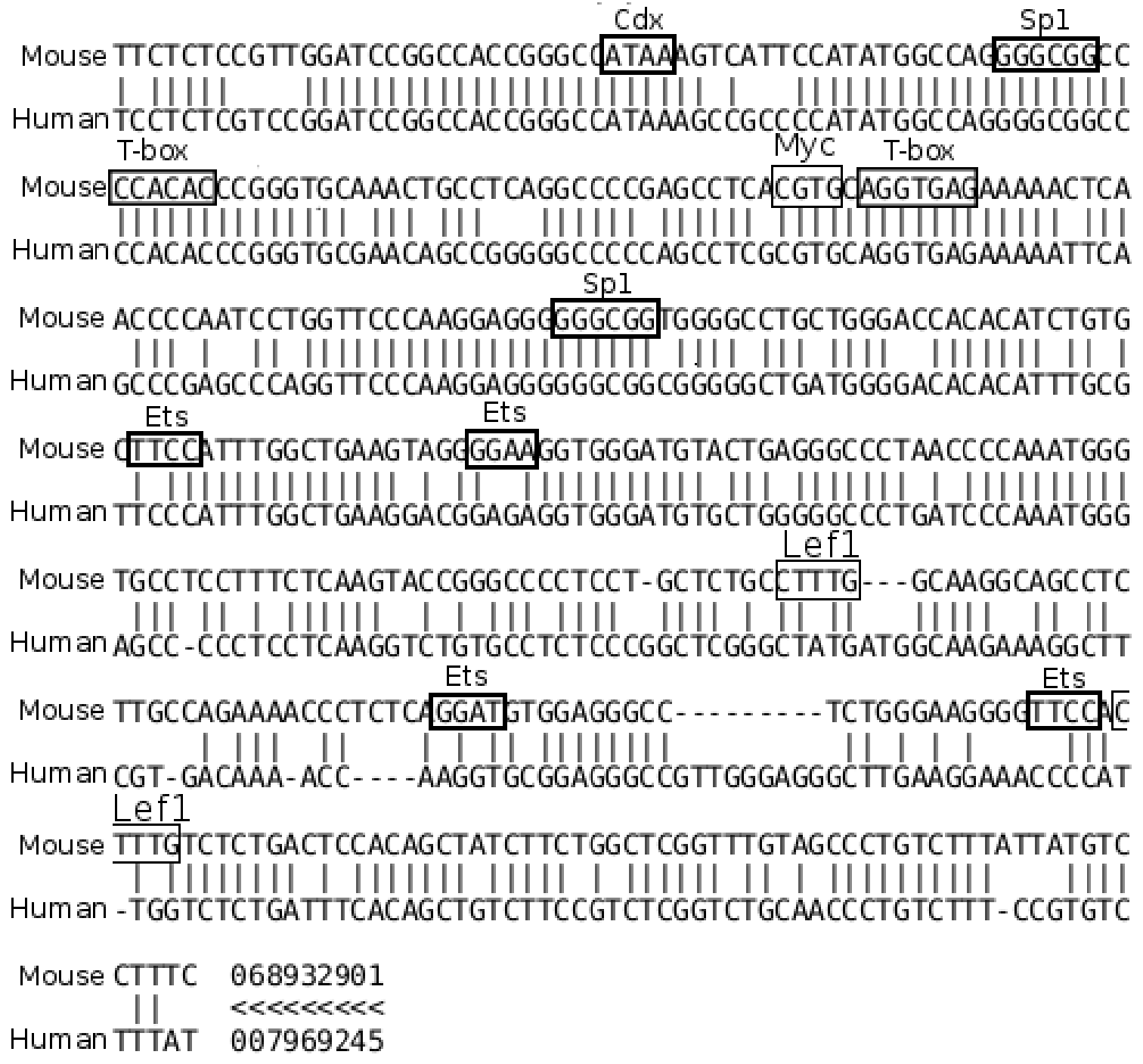

Supplement: Figure S1 — Sequence alignment of the region between −1.4 and −1.0 kb of the mouse Hes7 promoter (Top) with human (Bottom) and transcription factor binding sites (TFBS). Several conserved binding sites of transcription factors expressed in the PSM can be found. (TIF) [file pone.0053323.s001.tif]

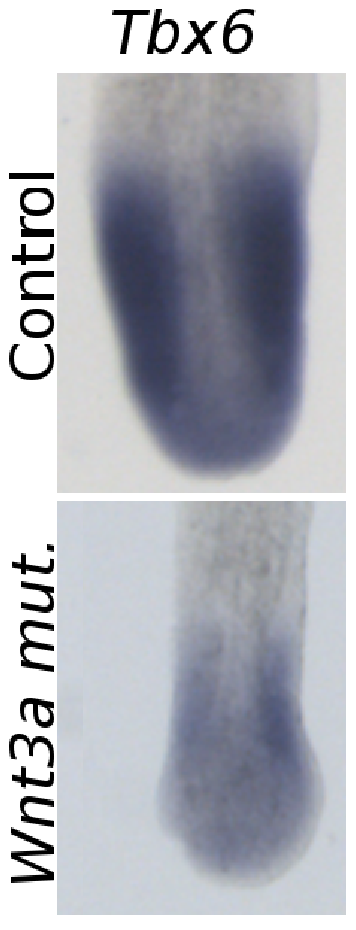

Supplement: Figure S2 — In situ hybridization of Tbx6 mRNA in E10.5 Wnt3a mutant embryos. Tbx6 expression is downregulated in the Wnt3a hypomorph embryos (Control: n = 2; Mutant: n = 2). (TIF) [file pone.0053323.s002.tif]

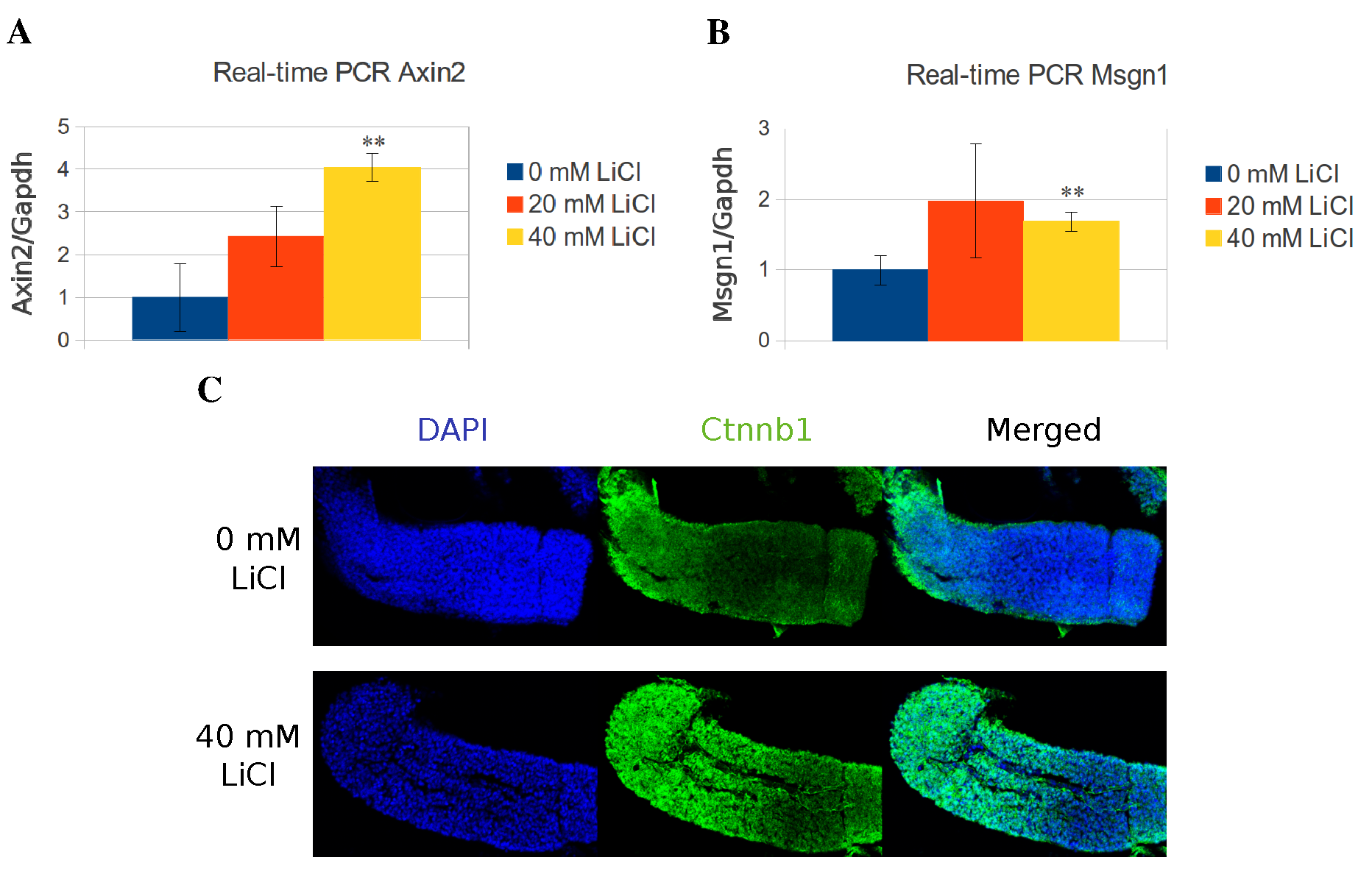

Supplement: Figure S3 — LiCl activates the Wnt pathway in the PSM. (A,B) E9.5 embryos were cultured with LiCl for 6 h and the expression of Axin2 and Msgn1 was measured by real-time PCR. The expression of both Axin2 (A) and Msgn1 (B) increases in the presence of 40 mM LiCl (0 mM: n = 10; 20 mM: n = 10; 40 mM: n = 10). (C) E10.5 embryos were cultured with 40 mM LiCl for 6 h and immunohistochemistry was carried with a Ctnnb1 antibody. Ctnnb1 protein is stabilized by 40 mM LiCl treatment (0 mM: n = 6; 40 mM: n = 8). (TIF) [file pone.0053323.s003.tif]

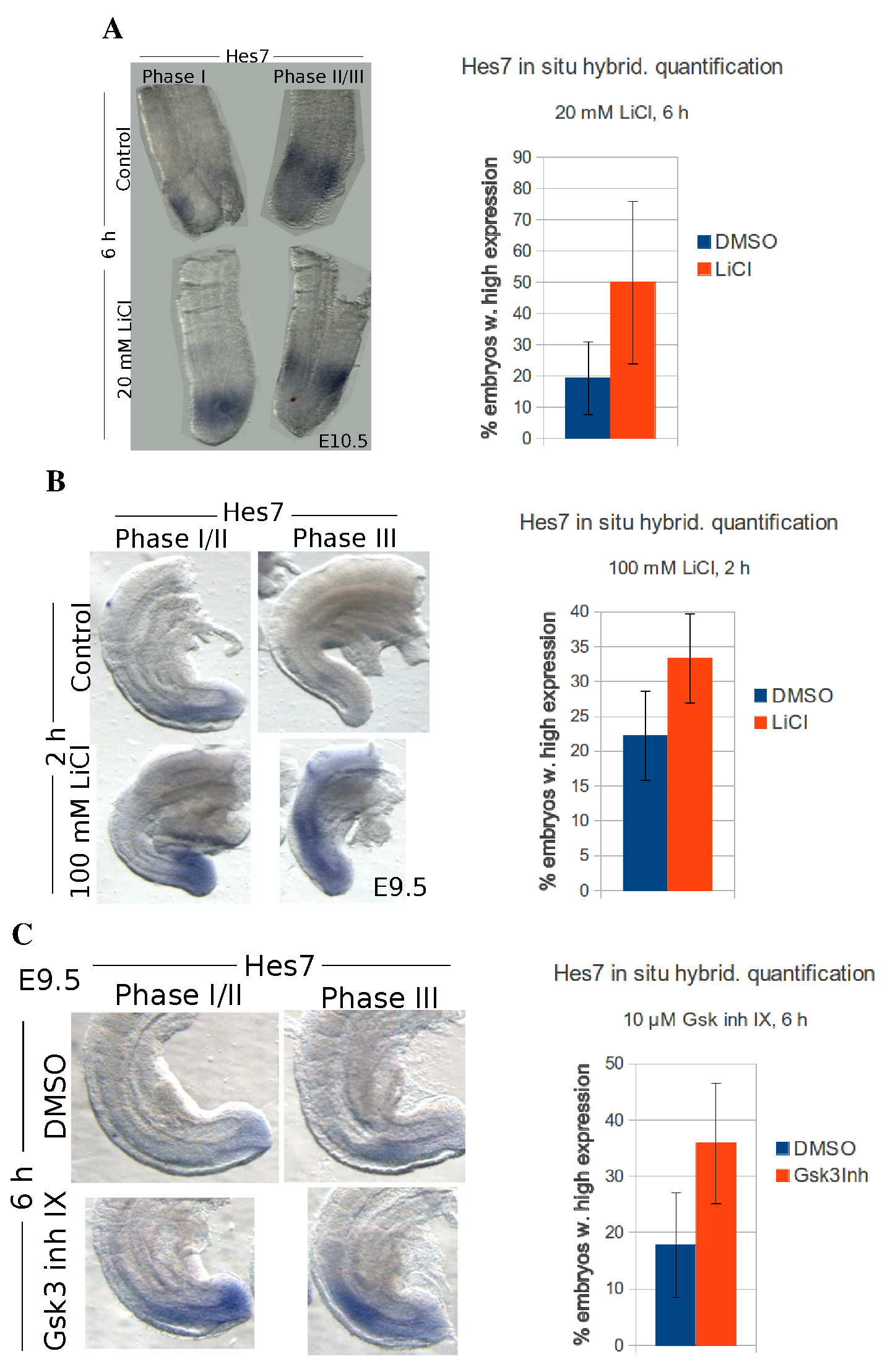

Supplement: Figure S4 — Hes7 in situ hybridization of embryos cultured in the presence of Gsk3 inhibitors. E10.5 embryos were cultured with 20 mM LiCl for 6 h (Control: n = 7; Treated: n = 10) (A), E9.5 embryos with 100 mM LiCl for 2 h (Control: n = 13; Treated: n = 14) (B) and E9.5 embryos with 10 µM Gsk3 Inhibitor IX for 6 h (Control: n = 13; Treated: n = 12) (C). Our results show a non-significant increase of the Hes7 expression levels under these culture conditions. (TIF) [file pone.0053323.s004.tif]

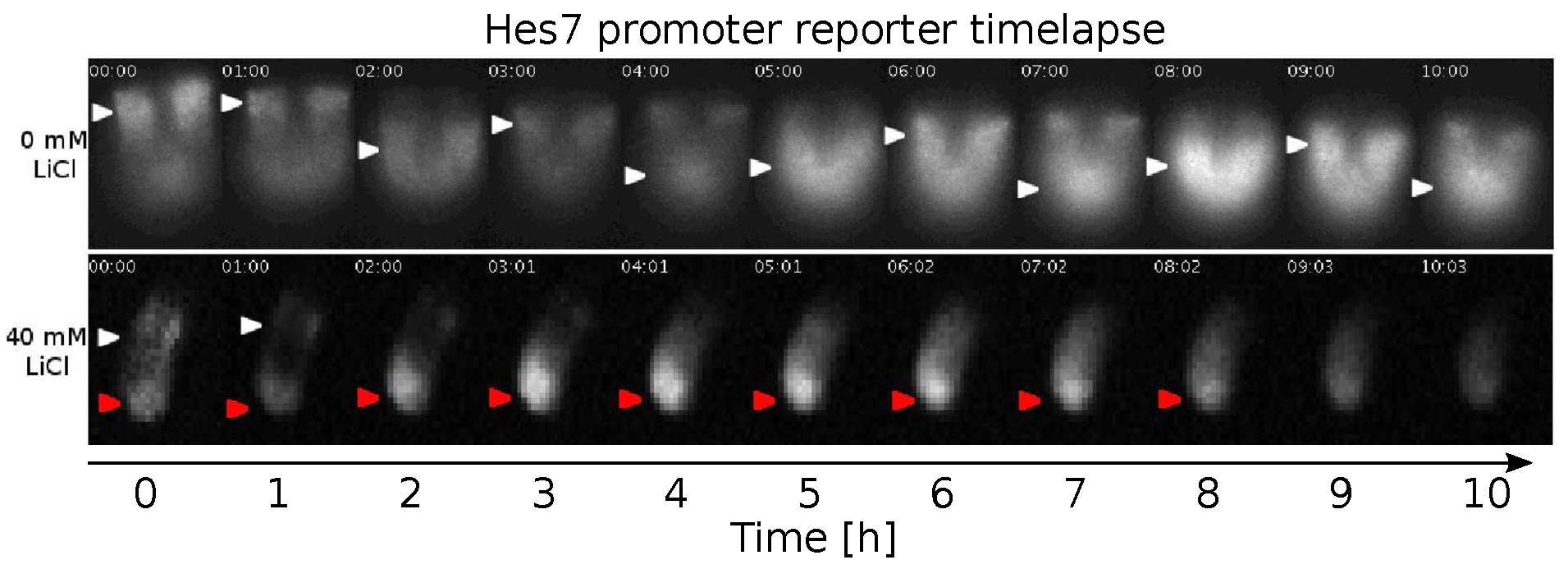

Supplement: Figure S5 — Timelapse imaging of E10.5 Hes7 promoter luciferase reporter embryos in the presence of the 40 mM LiCl. Some embryos treated with this concentration of LiCl showed stabilization of the Hes7 promoter reporter activity and arrest of oscillations (Red arrowheads). (TIF) [file pone.0053323.s005.tif]

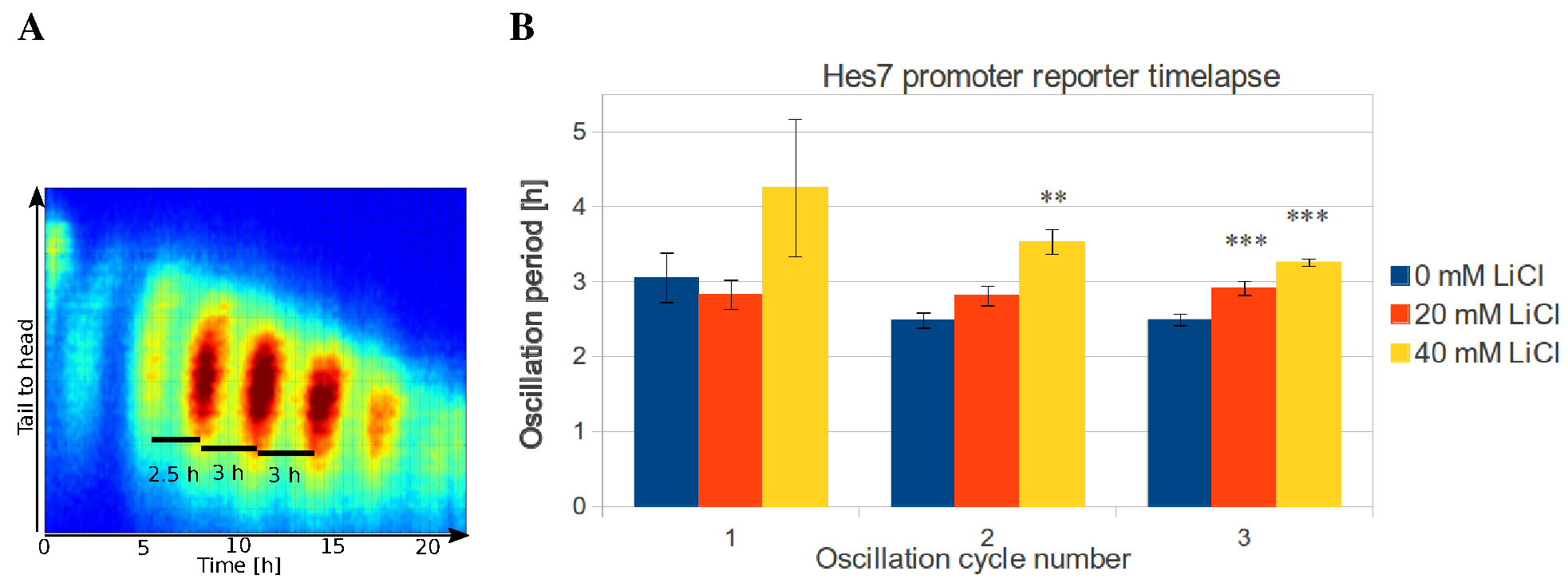

Supplement: Figure S6 — Period quantification method and duration of oscillation cycles after addition of LiCl. To measure the oscillatory period, we created a spatiotemporal plot of the timelapse activity with time in the x-axis and measured the distance between peaks in the posterior PSM (A). This allows us to measure the duration of individual cycles after addition of the chemical LiCl. We started to observe a period difference during the second oscillation cycle (B). (TIF) [file pone.0053323.s006.tif]

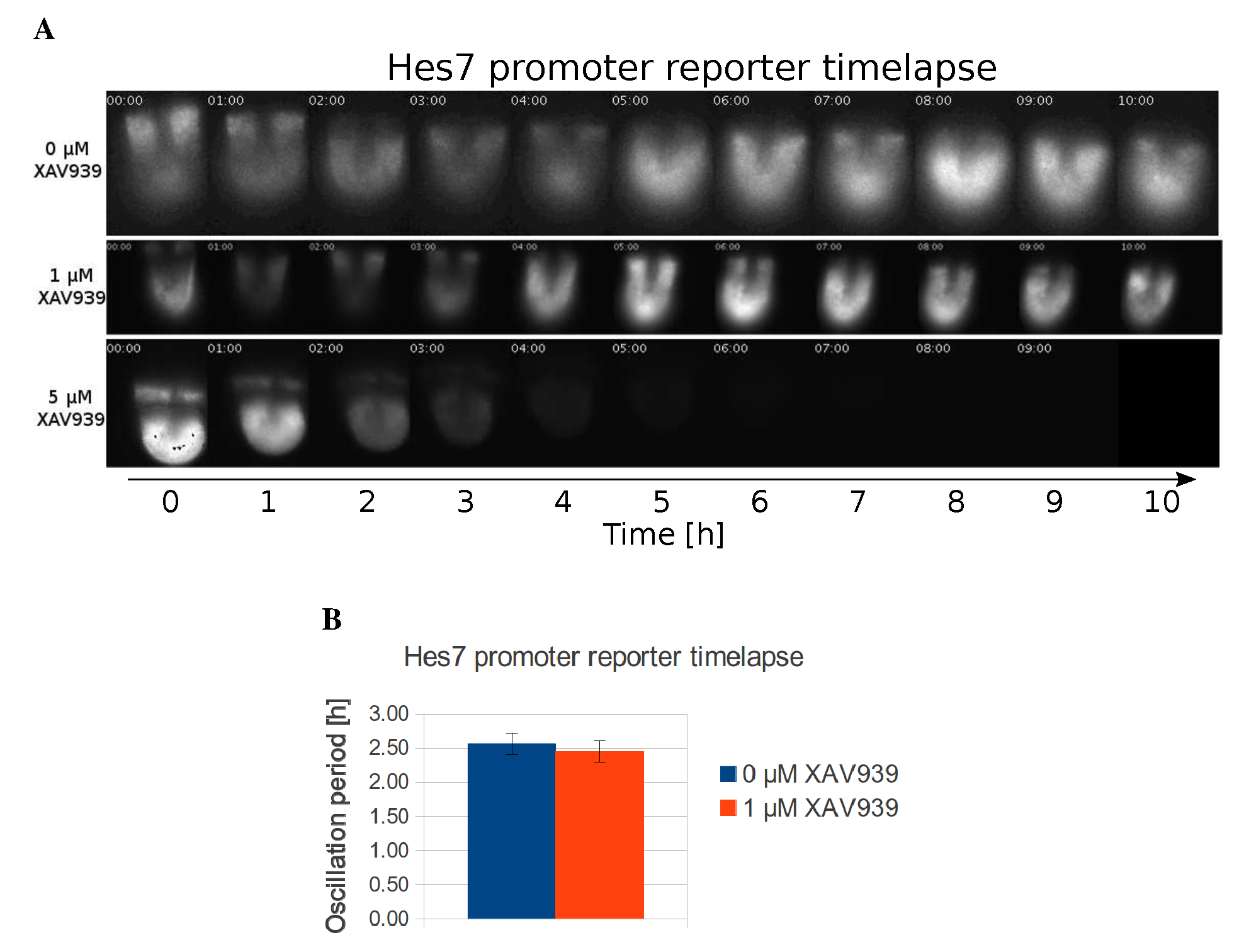

Supplement: Figure S7 — Timelapse imaging of E10.5 Hes7 promoter luciferase reporter embryos in the presence of the Wnt inhibitor XAV939 and period quantification. (A) The control and 1 µM XAV939 samples show the normal reporter activity, while 5 µM XAV939 treatment rapidly downregulates Hes7 promoter activity. (B) XAV939 treatment does not change the period between the control (n = 11) and the 1 µM XAV939 samples (n = 2). (TIF) [file pone.0053323.s007.tif]

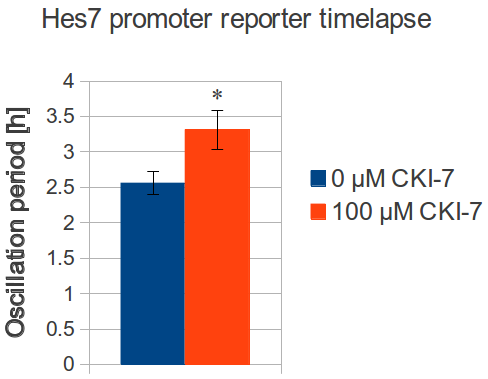

Supplement: Figure S8 — Period quantification of the oscillations of the E10.5 Hes7 promoter reporter embryos in the presence of the chemical agent CKI-7 observed by timelapse imaging. Treatment with 100 µM CKI-7 lengthens the period from 2.5 h (n = 11) to 3.3 h (n = 3). (TIF) [file pone.0053323.s008.tif]
